# Bile salt metabolism is not the only factor contributing to *Clostridioides* (*Clostridium*) *difficile* disease severity in the murine model of disease

**DOI:** 10.1080/19490976.2019.1678996

**Published:** 2019-12-02

**Authors:** Caitlin A. Jukes, Umer Zeeshan Ijaz, Anthony Buckley, Janice Spencer, June Irvine, Denise Candlish, Jia V. Li, Julian R. Marchesi, Gillian Douce

**Affiliations:** a Institute of Infection, Immunity and Inflammation, College of Veterinary Medical and Life Sciences, University of Glasgow, Glasgow, UK; b School of Engineering, College of Science and Engineering, University of Glasgow, Glasgow, UK; c Leeds Institute of Medical Research, Faculty of Medicine and Health, University of Leeds, Leeds, UK; d School of Health and Life Sciences, Glasgow Caledonian University, Glasgow, UK; e Faculty of Medicine, Department of Surgery & Cancer, Imperial College London, London, UK; f School of Biosciences, Cardiff University, Cardiff, UK

**Keywords:** Bile salt metabolism, *Clostridium difficile*, germination, antibiotics, disease severity

## Abstract

Susceptibility of patients to antibiotic-associated *C. difficile* disease is intimately associated with specific changes to gut microbiome composition. In particular, loss of microbes that modify bile salt acids (BSA) play a central role; primary bile acids stimulate spore germination whilst secondary bile acids limit *C. difficile* vegetative growth. To determine the relative contribution of bile salt (BS) metabolism on *C. difficile* disease severity, we treated mice with three combinations of antibiotics prior to infection. Mice given clindamycin alone became colonized but displayed no tissue pathology while severe disease, exemplified by weight loss and inflammatory tissue damage occurred in animals given a combination of five antibiotics and clindamycin. Animals given only the five antibiotic cocktails showed only transient colonization and no disease. *C. difficile* colonization was associated with a reduction in bacterial diversity, an inability to amplify bile salt hydrolase (BSH) sequences from fecal DNA and a relative increase in primary bile acids (pBA) in cecal lavages from infected mice. Further, the link between BSA modification and the microbiome was confirmed by the isolation of strains of *Lactobacillus murinus* that modified primary bile acids *in vitro*, thus preventing *C. difficile* germination. Interestingly, BSH activity did not correlate with disease severity which appeared linked to alternations in mucin, which may indirectly lead to increased exposure of the epithelial surface to inflammatory signals. These data confirm the role of microbial metabolic activity in protection of the gut and highlights the need for greater understanding the function of bacterial communities in disease prevention.

## Introduction

*Clostridiodes difficile* is a disease of the gut microbiome with onset associated with the use of antibiotics^1^ that reduce bacterial diversity within the colon. It is the leading cause of antibiotic-associated diarrhea (AAD) and the main cause of pseudomembranous colitis (PMC).^2^ Further, a significant number of patients suffer relapsing disease^3–^^5^ following cessation of antibiotics (metronidazole and vancomycin) used in treatment. Such episodes reflect persistent disruption of the microbiota, creating an environment in which the pathogen can flourish.^6,7^ Consequently, restoration of microbiota diversity has become the focus for new treatments including fecal microbiota transplantation (FMT),^8,9^ defined bacterial therapies^10–12^ and more recently sterile fecal filtrate transfer (FFT).^13^ Although these approaches frequently restore microbial diversity within the niche and eliminate disease, the long-term consequences of using undefined microbial therapies on long-term health, is not yet clear. This is relevant given the increasing burden of evidence that gut microbes play a role in diseases including diabetes, obesity and mental illness.^14^ These links have driven researchers to consider which organisms can and should be included in these preparations. Several groups have shown that specified cocktails of bacterial species may be sufficient to limit disease, at least in mice^11,15^ and whilst knowledge of the identity of key players is increasing,^11^ for such therapies to be successful and safe, it is important that we improve our understanding of the functional contribution offered by each organism.

In the context of *C. difficile* germination, one role performed by bacterial members of the gut microbiome is to modify bile acids (BAs). Primary bile salts produced in the liver are released in the small bowel and assist in the uptake of lipids and lipid-soluble vitamins from the diet. The breakdown of BAs is complex and involves several enzymes^16^ expressed by gut bacteria, the two most important in the context of *C. difficile*, being bile salt hydrolases (BSH) and the 7α-dehydroxylation (7αDH) enzymes.^17,18^ Modification is relevant as primary bile acid (pBA) taurocholic acid (TCA) induce spore germination whereas modified secondary bile acid (sBA), deoxycholic acid (DCA), are poor germinates and inhibit vegetative cell outgrowth.^19^–^21^ BSH are expressed by several members of the microbiota and are important for the removal of the amino acid sidechains, which allow other enzymes including the 7α-dehydroxylases to function (Supplementary Figure 1). This secondary process is more specialized and restricted to a few species including *Clostridium scindens, Clostridium hylemonae, Clostridium sordellii, Clostridium hiranonis* and various unclassified Eubacteria.^22,23^

**Figure 1.**
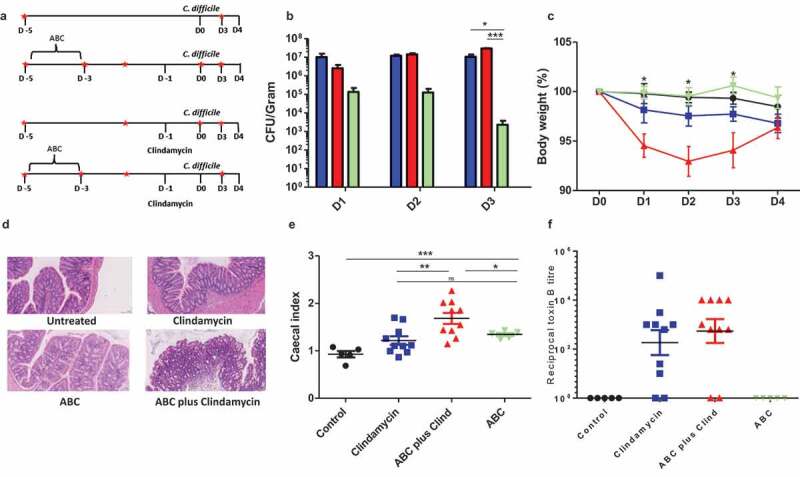
Antibiotic pre-treatment influences disease progression.

Multiple antibiotics have been associated with changes in BA metabolism including streptomycin,^24^ cefoperazone,^25,26^ clindamycin^26,27^, and vancomycin.^26,28^ The impact of disruption of BA metabolism and *C. difficile* disease has previously been demonstrated using *Cl. scindens*, which modifies cholic acid to produce DCA through the expression of a 7α-dehydroxylase. Treatment of *C. difficile* infected mice with this strain-reduced mortality but treatment was only fully protective when *Cl. scindens* was combined with three other bacteria.^11^ This result suggests that other metabolic functions may be important in germination, colonization resistance, and disease severity.

Until recently, one factor limiting progress was the availability of an animal model that was tractable to *C. difficile* infection and in which knowledge of the microbiome was significant. Although the Golden Syrian Hamster is recognized as the standard model for the study of acute toxin-mediated *C. difficile* disease, the rapid and fatal nature of infection limits the study of changes to gut microbiome composition. However, two mouse models, described in recent years have revealed that pre-treatment with different combinations of antibiotics prior to infection, can influence disease outcome. For example, treatment with clindamycin alone can render mice susceptible to transient colonization with high numbers of *C. difficile*, which are then suppressed by gut microbiome reestablishment.^15^ Interestingly, these animals remain asymptomatic but persistently infected with low (almost undetectable) numbers of bacteria, which proliferate if microbiota diversity is modified by further antibiotic treatment. In contrast, Chen *et al*., (2008)^29^ showed that treatment with a cocktail of five antibiotics (colistin, metronidazole, vancomycin kanamycin, and gentamicin; the ABC cocktail) prior to clindamycin (ABC+clindamycin) resulted in acute disease.^29^ Animals lose weight, develop loose feces and show significant gut pathology 2–3 days post-infection. Differences, especially the degree of epithelial damage and tissue disruption are significant and probably reflect the virulence of *C. difficile* strains and the diversity of gut microbiome of the individual mouse.

To determine whether disease outcome reflected changes in the composition and metabolic functional activity of the gut microbiome, we compared animals pre-treated with different antibiotic regimens prior to infection. Using 16S rRNA gene analysis (metataxonomics), amplification of sequences encoding the BSH enzymes and direct measurement of the abundance of bile salts, we hoped to determine if the extent of bile salt modification influenced disease severity.

## Results

The impact of different antibiotic regimens on disease severity was determined by treating animals as described (Figure 1(a)). These animals were subsequently orally challenged with 1 × 10^5^ *C. difficile* B-I7 spores and animals monitored for changes in behavior, appearance, and weight loss; animals showing a drop in weight of greater than 15% were culled. Colonization was determined by the recovery and quantification of *C. difficile* from fresh fecal material. Using these critertium, untreated, but infected animals showed no weight loss and failed to become colonized. Treatment with the ABC cocktail alone resulted in transient colonization with significantly lower numbers of both spores and vegetative cells of *C. difficile* detected in the feces compared to clindamycin treatment (*p* = .0107) or ABC plus clindamycin treatment (*p* = .0074) at 3DPI (days post-infection) (Figure 1(b)). High bacterial loads (spores and vegetative cells) were recorded for animals given clindamycin, either alone or in combination with ABC, in both the feces (3DPI) and from cecal and colonic tissue at the time of cull (4DPI) (Supplementary Figure S2). Significant weight loss was noted between infected animals pretreated with ABC+clindamycin and untreated animals (*p* < .05) but not animals treated with any other antibiotic combination (Figure 1(c)). This result correlated with observations of cecal and colonic tissue damage compared to those given either ABC or clindamycin alone (Figure 1(d)). Furthermore, at the time of cull (4DPI) the cecal index (proportion of the body weight contributed by the cecum), an indirect measurement of inflammation, was significantly raised when animals were treated with either combination of antibiotic. Interestingly, combining antibiotic treatments further significantly enhanced the cecal index compared to ABC or clindamycin alone treated mice (Figure 1(e)). As Toxin A and Toxin B produced by the bacteria are associated with disease, functional activity of filtered cecal and colonic contents from infected mice were tested. Surprisingly, differences in toxin activity between these groups did not correlate with pathology (Representative Toxin B data is shown in Figure 1(f); Toxin B activity in colon samples; Toxin A data not shown). From this data, we determined that disease severity was influenced by a non-toxin related modification to the niche.

## Impact of antibiotic treatment on gut microbiome community structure and function

To determine if severity could be linked to changes in microbial gut composition, a metataxonomic analysis was performed on DNA extracted from fecal samples collected during the experiment (denoted by red stars in Figure 1(a)). Interestingly, fecal material from animals treated with ABC+clindamycin appeared associated with visually greater amounts of mucus observed during fecal collection. Further, a substantial mucus layer was observed following the centrifugation of this material.

**Figure 2.**
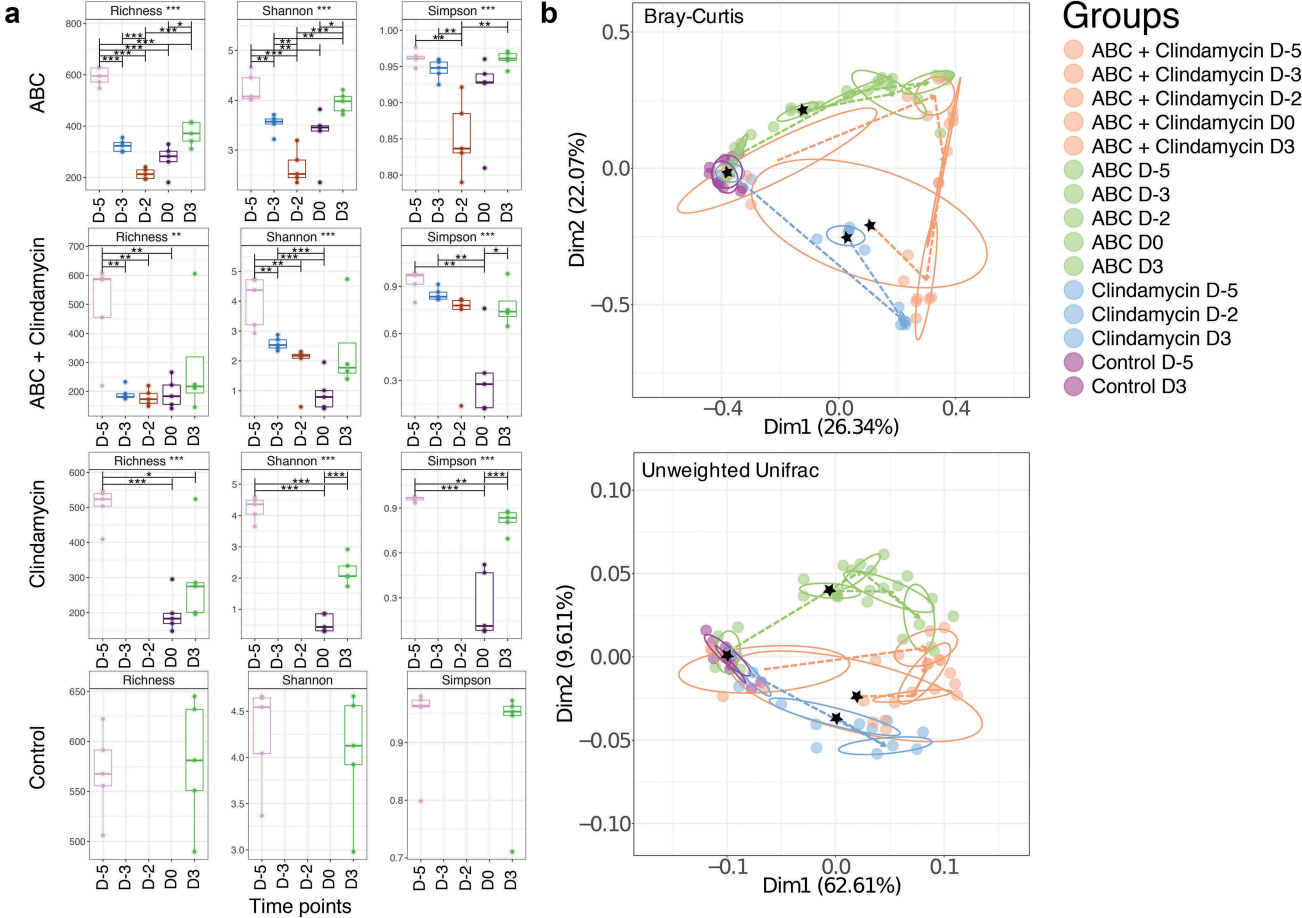
Analysis of alpha and beta diversity from different antibiotic treatment groups.

Using three independent, but complementary bioinformatic methods, the alpha and beta diversity of the samples were assessed. This revealed that mice treated with clindamycin or the ABC+clindamycin cocktail showed the greatest microbial changes in the gut microbiome (Figure 2(a)). These changes were less apparent 3DPI, reflecting a recovery of the composition of the gut microbiomes. A PCoA Cluster analysis of these samples revealed congruence of samples prior to antibiotic treatment suggesting a similarity in the structure of the bacterial communities in the gut microbiomes within these animals (Figure 2(b)). Post-antibiotic treatment (D0) clustering was distinct between animals treated with ABC alone and those given clindamycin treatment or ABC+clindamycin. We concluded that significant changes in bacterial community diversity were dependent on antibiotic treatment.

**Figure 3.**
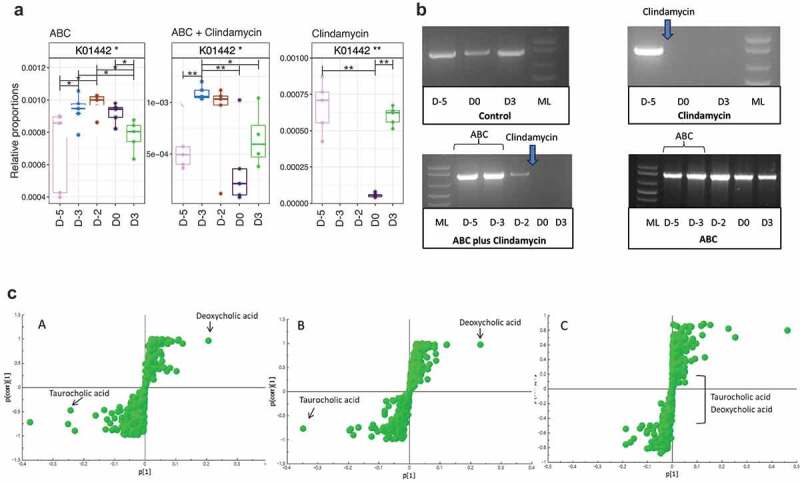
The impact of different antibiotic regimes on the amplification of DNA encoding BSH and abundance of primary and secondary BA.

As changes to BA metabolism and *C. difficile* infection have been described previously^11^ we indirectly analyzed metabolic functions in the gut microbiome to determine if genes encoding these enzymes could predict infection susceptibility. Using Tax4fun,^30^ which bioinformatically infers the metabolic capability of the community based on predictions of microbiome members from metataxonomic analysis, the abundance of sequences encoding the 7α-dehydroxylation enzymes, involved in secondary metabolism of BA, were studied. Apparent absence of these sequences may reflect the relatively low percentage of organisms expressing this enzyme. In contrast, work focusing on BSH, the gateway enzyme for the pathway, showed BSH sequences were reduced in mice treated with clindamycin, or ABC+clindamycin (Figure 3(a), timepoint D0, post-clindamycin treatment). In contrast, a reduction at this timepoint was not observed in animals given the ABC cocktail alone. To confirm this observation, degenerate PCR primers that target BSH genes were used to amplify these sequences from fecal DNA samples. Bands of approximately 850 bp (subsequently sequenced and confirmed as BSH) were amplified from samples taken prior to treatment with clindamycin, from animals not receiving antibiotics or those given the ABC cocktail alone (Figure 3(b)) but not from those post-clindamycin treatment. A second, alternative set of degenerate BSH primers confirmed this observation (data not shown). Thus, we concluded that clindamycin treatment was sufficient to eliminate members of the microbiota that influenced BA modification through BSH activity. Time of flight (TOF) Mass spectrometry analysis of BAs extracted from the cecum of mice confirmed the relative abundance of TCA and DCA in samples from infected animals (Figure 3(c)). Comparison of single fecal ions from cecal extracts revealed that treatment with clindamycin was sufficient to modify the relative abundencies of BA (summary of bile salts analyzed is provided in supplementary Figure 3). In particular, high levels of TCA were linked with clindamycin treatment (Figure 3(c-a, c-b); either alone or in combination with ABC). In contrast, no differences in BA abundance were observed between the clindamycin and the ABC+clindamycin treated groups (Figure 3(c-c)).

## Role of bile salt modification on *C. difficile* spore germination and vegetative growth

**Figure 4.**
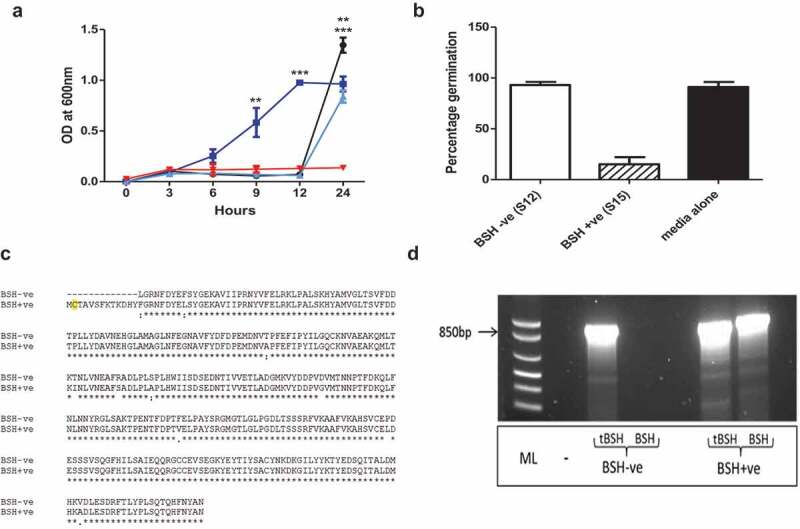
Impact of BA’s on spore germination and outgrowth.

To confirm the impact of different BAs on BI-7 spore germination, spores were inoculated into growth medium containing 0.1% TCA, CA, or DCA, respectively (Figure 4(a)). As expected, spores exposed to TCA germinated rapidly reaching the stationary phase by 12 h, confirming TCA induces germination. In contrast, equivalent numbers of spores in BHI or BHI+CA, showed a delay in germination and outgrowth (+12h), indicating that CA is a less effective germinant. No growth was observed in the culture which included DCA, confirming that DCA inhibits the vegetative outgrowth of *C. difficile*.

To directly confirm the link between TCA modification and the microbiome, several bacterial species were cultured from fecal material. To identify those strains capable of TCA modification, isolates were grown in medium containing 0.1% TCA. Spent media was then filtered to remove bacteria and the resultant filtrate inoculated with *C. difficile* spores. Whilst several isolates were unable to modify TCA and thus prevent *C. difficile* germination, two strains of *Lactobacillus murinus* were isolated with contrasting capacity to limit (S15) or allow TCA induced spore germination (S12) (Figure 4(b)) were identified. To determine if spore germination was related to BSH activity, the genomes of both strains were sequenced and BSH-like sequences annotated. This analysis confirmed that BSH was encoded by both strains, however, in S12, 39 nucleotides were missing from the start of the annotated BSH gene, including a key cysteine residue, essential for BSH activity^16^ and the methionine start codon (Figure 4(c)). This truncation was verified using primers designed to amplify the truncated (939 bp) or full-length BSH gene (978 bp), respectively (Figure 4(d)). This observation highlights for the first time that the functional activity of BSH may vary between isolates of the same strain recovered from the microbiome. This is relevant given the search for defined combinations of bacteria that have efficacy for *C. difficile* treatment. In particular, it highlights the need for careful characterization of strains especially when assigning generic activities to particular strains of bacteria.

## Impact of changes in the diversity of the microbiome and disease severity

**Figure 5.**
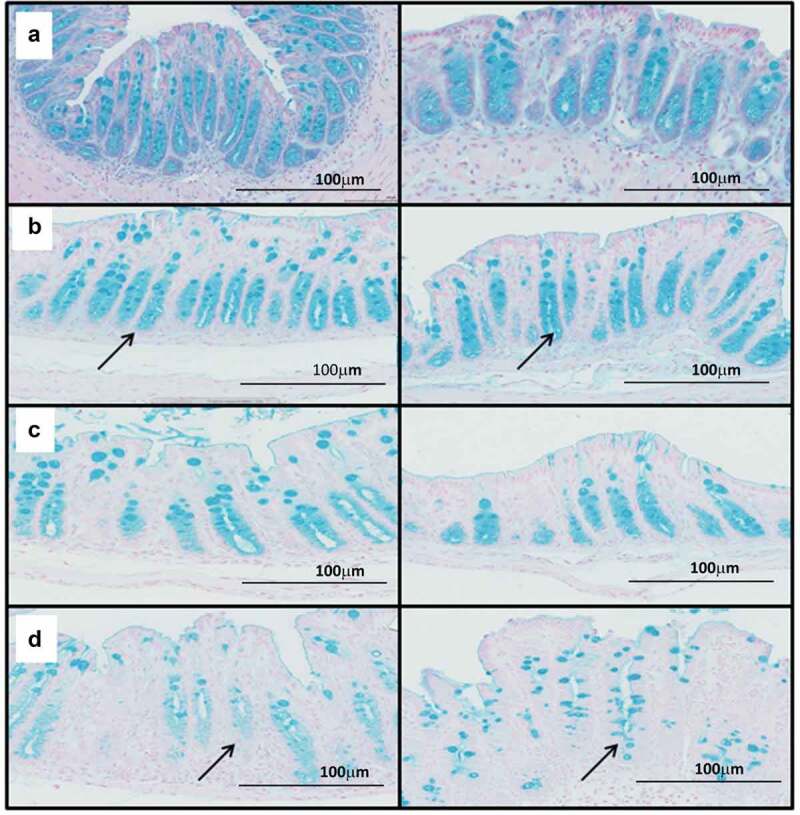
Modifications to mucin in mice treated with different antibiotic combinations.

Whilst this evidence supports and extends knowledge of BA metabolism and colonization resistance, it failed to establish why animals treated with ABC+clindamycin develop acute disease. To explore this discrepancy, differences in the bacterial species were compared between animals pre-treated with clindamycin and those given clindamycin+ABC. This revealed a significant reduction in species that are normally associated with mucus colonization with a concurrent increase in mucus degrading species (Tables 1 and 2 and Supplementary Table 1). These data suggested that disease severity may be linked to changes to the mucus barrier leading to increased epithelial exposure to both *C. difficile* and its toxins. To explore this hypothesis, cecal and colonic tissue from infected mice were stained with Alcian blue, which binds to acidic polysaccharides, including mucopolysaccharides. This showed mucus was concentrated in the crypt regions of tissue in untreated and uninfected animals (Figure 5(a)), which was largely unaffected by infection (Figure 5(b)). In contrast, the crypt mucus appeared reduced in infected animals treated with ABC+clindamycin (Figure 5(d)) compared to those given clindamycin alone (Figure 5(c)). These results led us to conclude that modification to the bacterial complexity of the gut microbiome affects mucus integrity. The consequential increase in OTUs associated with mucin degradation and loss of mucin dwellers may reflect these changes.

**Table 1.** Significantly increased OTU representation in samples from mice treated with ABC+Clindamycin compared to clindamycin alone at D0.

*Significantly Increased OTUs*	Adjusted *P* value
*OTU_780 Parabacteroides*	1.77E-18
*OTU_ 11 Parabacteroides goldsteinii*	2.55E-18
*OTU_ 3 Parabacteroides distasonis*	2.51E-15
***OTU_ 2 Bacteroides thetaiotaomicron***	**1.76E-14**
*OTU_ 784 Parabacteroides*	9.94E-14
*OTU_772 Parabacteroides*	3.52E-13
*OTU_34 Firmicutes*	1.18E-10
*OTU_812 Parabacteroides*	2.92E-09
*OTU_ 22 Erysipelotrichaceae bacterium Alo17*	7.83E-09
*OTU_850 Parabacteroides*	2.63E-08
*OTU_468 Bacteroides*	2.92E-08
*OTU_24 Bacteroides intestinalis*	3.07E-08
*OTU_7 Enterococcus*	1.15E-07
*OTU_5 Allobaculum*	1.25E-07
*OTU_10 Barnesiella*	3.44E-07
*OTU_122 Unclassified bacteria*	2.04E-05
*OTU_40 Porphyromonadaceae*	2.04E-05
*OTU_17 Burkholderiales*	2.65E-05
*OTU_733 Parabacteroides distasonis*	3.91E-05
*OTU_755 Parabacteroides*	3.95E-05
*OTU_750 Parabacteroides*	5.14E-05
*OTU_734 Parabacteroides*	5.25E-05
*OTU_756 Parabacteroides*	8.68E-05
*OTU_ 64 Betaproteobacteria*	9.65E-05

**Table 2.** Significantly decreased OTU representation in samples from mice treated with ABC+Clindamycin compared to clindamycin alone at D0.

*Significantly decreased OTUs*	Adjusted *P* value
*OTU_15 Lactobacillus johnsonii*	7.87E-32
*OTU_ 36 Porphyromonadaceae*	8.14E-12
*OTU_223 Lactococcus*	2.37E-11
*OTU_21 Enterobacteriaceae*	2.31E-10
*OTU_45 Lactobacillus intestinali*	2.92E-09
***OTU_91 Mucispirillum schaedleri***	**2.92E-09**
***OTU_181 Enterorhabdus mucosicola***	**1.24E-05**
*OTU_53 Porphyromonadaceae*	2.60E-05
*OTU_47 Odoribacter*	3.95E-05
*OTU_88 Lachnospiraceae*	5.0929e−05

## Discussion

The availability of immunological tools and transgenic animals have made mice increasingly the model of choice for the study of *C. difficile* disease. The transition to the mouse from the hamster has been achieved through the identification of antibiotic regimens that allow for the study of both colonization and disease.^15,29^ In this study, we sought to understand the basis of differences in disease outcome when animals were challenged with the epidemic 027 strain BI-7. Whilst animals showed the same patterns of disease as reported elsewhere (weight loss and tissue damage in the ABC+clindamycin group and transient colonization in animals treated with clindamycin alone), no mouse mortality was observed. Their survival contrasts with studies using VP10463, which is a naturally high toxin producer. This difference may reflect the amount of toxin produced *in vivo* or the protective influence of the bacterial gut microbiome communities in mice sourced from different commercial providers.

The evidence that bile metabolism influences *C. difficile* infection is now significant both *in vitro*^31^ and *in vivo*,^26^ although the extent to which secondary BA influence germination and outgrowth varies between clinical isolates.^32^ Consequently, attention has focused on the relative abundance of BSH and 7α-dehydroxylase genes within gut samples following antibiotic treatment. This study reveals that organisms encoding BSH genes were largely eliminated by clindamycin treatment alone. This change directly correlated with alternations to TCA and DCA concentrations, with high level of TCA found in the caecums of mice from which high numbers of *C. difficile* were recovered. Further isolation of *L. murinis* strains from animals pretreatment allowed confirmation that BSH activity influenced *C. difficile* germination at least *in vitro*. Sadly, the capacity to determine if these strains could prevent disease *in vivo* was hampered by their sensitivity to clindamycin. However, the observation that clindamycin treatment alone eliminated BSH activity is significant given that, a decrease in 10 OTU’s that encode 67.3% of the BSH genes in patients with recurrent infection compared to controls, has been described.^18^

Although BSH carry out the gateway reaction for bile metabolism, 7α-dehydroxylation is also an important part of the pathway that generates DCA. As shown here, CA does not induce spore germination and conversion of TCA to CA by a BSH producing *L. murinus* can reduce spore germination *in vitro*. The cleavage of the amino acid from the BA by the action of BSH results in the release of taurine, which can be reduced to H_2_S. H_2_S is an essential substrate for 7α-dehydroxylase producing bacteria, suggesting that these bacterial populations act synergistically.^33^ In fact, recovery of BSH activity has been linked to increased 7α-dehydroxylation activity within the gut microbiome of rats following clindamycin treatment.^34^ In addition, a significant increase in Clostridial species, DCA and 7α-dehydroxylation activity was observed in mice fed CA.^35,36^ These studies strengthen the hypothesis that restoration of bile metabolism through the introduction of BSH producing bacteria, or through feeding of CA, is achievable. It is feasible that many of the therapeutic bacterial mixtures already contain strains expressing BSH activity.^10,15^ In fact, the successful cocktail used to treat mice by Lawley *et al*., (2012) contained a BSH producer we have identified in other unpublished work. Further, the bacteriotherapy, ‘rePOOPulate’, a mixture of 33 bacterial isolates used to treat patients, contains several potential BSH producers as well as bacteria that may be capable of 7α-dehydroxylation including several Clostridia and *Eubacterium*. These observations hint at least one mechanism by which these formulations may work. In this study, attempts to investigate the 7α-dehydroxylation enzyme were unsuccessful. However, metataxonomic analysis of the microbiota pre- and post-clindamycin treatment did reveal a significant decrease in OTU_69, which is a member of the *Clostridium* XIVa cluster known to carry out this function; the loss of which has been linked to decreased colonization of *C. difficile* within the mouse model.^37^

From these data, we conclude that the observed disease severity, between these two different mouse models, is not pathogen driven, as no difference in the levels of colonization or toxin production were observed between treatment groups. This contrasts with reports that suggest that BA derivatives such as methyl cholate have a direct impact on toxin actvity.^38^ In our hands, increased tissue pathology was a consequence of antibiotic-associated modification of the microbiota, directly influencing the environmental context of the niche and survival and growth of both beneficial and harmful organisms. Whilst the use of clindamycin was associated with a bloom of *Enterobacteriaceae*^39,40^ addition of the ABC cocktail was linked to changes in mucin associated organisms. In particular, an increase in *Bacteroides thetaiotaomicron* a specialized mucin degrader^41^ and the loss of three mucus dwelling species, *Mucispirillum schaedleri, Enterohabdus mucosicola*, and *Lactobacillus intestinalis*. Further, evidence that mucus degradation plays a role in disease severity has been provided by human intestinal organoids, which showed that *C. diffficile* was associated with both reduction of MUC2 production, which, along with increased mucin degradation, resulted in a thinner mucin layer.^42^ *In vivo*, animals treated with metronidazole (one of the five antibiotics included in the ABC cocktail) and infected with *Citrobacter rodentium* showed increased disease severity which was linked to reduced mucin production.^43^ The implication of disruption to mucus layer homeostasis is that the epithelial surface will be highly exposed to *C. difficile*, its toxins (TcdA, TcdB, and CDT) and to components of the gut microflora associated with inflammatory signaling. The increased disease associated with the ABC+clindamycin probably reflects a combination of all three factors.

Changes to the nutritional environment as a consequence of dysbiosis, induced by antibiotic treatment, has also been implicated in CDI severity. In particular, loss of many primary fermenters, such as *Lachnospiridae* and *Lactobacillus* (significantly reduced in this study) generates a niche in which alternative primary fermenters organisms such as *B. thetaiotaomicron to* proliferate, producing alternative endproducts of fermentation, such as succinate that can be utilised by *C. difficile* as a carbon source.^44,45^ In addition, sialic acid released following mucus degradation can also be utilized by *C. difficile* for growth.^46^ Further, in mono-associated rat studies, *B. thetaiotaomicron* has been shown to increase goblet cell differentiation and expression of mucus utilization genes. In these studies, this change was ameliorated by administering *Faecalibacterium prausnitzii* which restored homeostasis to this system. The implication from this work is that these organisms work synergistically to regulate the goblet cell generation and mucin glycosylation.^47^

In addition to alterations in the abundance of mucus-associated bacteria, ABC+clindamycin treatment affected the number of species that belong to the *Parabacteroides* genus, including *P. goldsteinii* and *P. distasonis*. This observation is relevant as mice with increased numbers of *Parabacteroides* and *Bacteroides* have a more diffuse mucus layer^48^ which may explain the increased quantity of mucus observed in mouse feces. Increased representation of *Parabacteroides* has also been associated with mice that shed persistently high numbers of spores^15^ and also with mice that succumb to *C. difficile* infection.^37^ Similarly, increases in *Parabacteroides* have been noted in humans infected with *C. difficile* suggesting either a role for these bacteria in infection^6,49^ or the creation of a nutritional niche in which they can flourish.

These data are in agreement with several other studies that have sought to understand the impact of different antibiotics on colonization resistance to *C. difficile*.^11,26,50^ Although different combinations of antibiotics have been tested, the importance of multiple assemblies of bacteria including *Porphyromonadaceae, Lachnospiraceae, Lactobacillus*, and *Alistipes*, in limiting *C. difficile* growth is clear. Whilst organisms capable of modifying bile acids offer one mechanism of control, maintaining competition for nutrition and energy offer a second. The capacity of *C. difficile* to directly exploit the restructured nutritional landscape as a consequence of antibiotic treatment has recently been explored in mice using a genome-scale metabolic model with a transcriptome-enabled metabolite scoring algorithm.^51^ This work revealed that *C. difficile* ferment amino acids preferentially, but are also capable of using host-derived glycan components as sources of energy. This ability to exploit the environmental changes helps explains why *C. difficile* is so effective at exploiting the modified niche. Whilst changes in response to antibiotics may vary from person to person, increased knowledge of the functional contribution of different populations will become increasingly important, especially when treating vulnerable patients. Furthermore, these data offer microbial genes as potential markers of disease susceptibility. For example, BSH in this study serves as a good marker for healthy bile metabolism, additionally, relative proportions of mucus degrading enzymes offer a good indicator of disease susceptibility. Future work will help establish if the representation of these markers could help predict vulnerability to *C. difficile* disease.

## Materials and methods

### Bacterial strains

*C. difficile* strains BI-7 (genotype 027/BI, Toxinotype III; clindamycin^R^, thiamphenicol^R^, erythromycin^S^, tetracycline^S^, ciprofloxacin^R^, vancomycin^S^) used in this study was a kind gift from Dale Gerding.

### Animal models

All procedures were performed in strict accordance with the Animals (Scientific Procedures) Act 1986 with specific approval granted by the Home Office, UK (PPL60/4218). Food and water were provided *ad libitum* and animals kept at a constant room temperature of 20–22°C with a 12-h light/dark cycle. Groups of five C57/bl6 mice aged 6–8 weeks supplied by Charles River (Edinburgh) were used in each treatment group. The antibiotic cocktail was administered *ad libitum* in the drinking water as previously described^29^ with clindamycin given at (150 mg/Kg), by oral gavage 2 days after the cessation of the antibiotic cocktail. Animals were each challenged with 10^5^ spores of *C. difficile* BI-7 1 day after clindamycin treatment. Mice were monitored closely post-infection and weighed daily to determine the severity of the disease. Animals with a weight loss greater than 15% were culled.

### C. difficile *shedding and organ colonization*

Fresh fecal samples collected daily were weighed, serially diluted in PBS and cultured on Braziers cycloserine, cefatoxaine, egg yolk (CCEY) agar at 37°C for 48 h. 4 days post-infection animals were culled and the cecum and colon harvested. Enumeration of total counts and spore-specific counts in lumen associated (LA) material and tissue associated (TA) were performed as previously described.^52^

### DNA extraction from feces – samples for microbiome analyses

Multiple fresh fecal samples from individual animals were collected from animals prior to ABC treatment (D-5), immediately post ABC treatment (D-3) but post ABC prior to clindamycin treatment (D-1) post-clindamycin treatment (D 0) and 3 days post-infection 3DPI (D3). Fecal samples were immediately processed using the FastDNA^TM^ Spin kit for soil as to the manufacturer’s instructions. DNA was stored at −80°C until required.

### Toxin detection

Toxin assays were carried out as described previously^52^ on filtered luminal content was collected from the cecum and colon of infected mice. Monolayers of Vero cells (kidney epithelial cells) and HT-29 cells were used to evaluate Toxin B and Toxin A activity, respectively. Results are expressed as the reciprocal of the final dilution of supernatant that caused cell rounding.^52^

### Histology staining

Tissue samples were harvested from the cecum and colon at postmortem and were immediately fixed in 10% formalin. Embedded tissue sections were cut and stained with hematoxylin and eosin^53^ or Alcian Blue.

### Cecal index as an indirect measurement of inflammation

Cecal index was calculated by determining the percentage of body weight that was made up by the cecum. The organ was washed and the content removed before weighing. Increased cecal weight is a potential indicator of organ thickening and edema that has been linked to increased inflammation.

### Amplification of V4 region of 16S rRNA gene

The V4 regions of the 16S rRNA gene were amplified and sequenced using the Illumina Miseq platform. For each sample triplicate 25 µl PCR reactions were carried out containing 12.5 µl of Phusion high fidelity master mix, 0.87 µl of Universal forward primer, 0.87 µl of indexed reverse primer, 1.25 µl of DMSO, 6.5 µl of nuclease-free water and 3 µl of DNA (2 ng). Primers contained a 12 bp unique identifying Golay Tag to allow multiplexing of samples (supplementary Table 2). Cycling conditions were 95°C for 5 min followed by 25 cycles at 95°C for 20 s, 60°C for 15 s and 72°C for 40 s followed by a final 72°C extension for 10 min. Replicates were pooled and gel purified using a Qiagen gel purification kit as to the manufacturer’s instructions. Library preparation and sample sequencing were performed at the Liverpool Center for Genomic Research.

#### Availability of supporting data

The sequencing data are available on the European Nucleotide Archive under the study accession number: PRJEB34878 (http://www.ebi.ac.uk/ena/data/view/PRJEB34878).

### Preparation of spores for germination assay

*C. difficile* strains were maintained as spore stocks at −80°C. Spore preparation was carried out as described previously.^52^

### Germination assays

Assays were carried out in triplicate with a minimum of three biological replicates for each sample. BHI was used as a negative control, BHI with 0.1% TCA as a positive control of maximal germination. BHI containing 0.1% CA or DCA were also included to confirm their role in germination.

To determine if the two *L. murinus* strains were able to modify BA’s directly, 30 µl of overnight culture grown in BHI was used to seed 3 ml of BHI containing 0.1% TCA. The culture was grown anaerobically for 24 h and culture supernatants sterilized using a 0.2 μm Ministart® syringe filter (Sartorius) to remove bacteria from the media. The pH of the filtered supernatant was adjusted to 7 and the media anaerobically conditioned for 2 h before the addition of 1x10^4^/ml pre-prepared spores (whose number was determined by inoculation onto BHI plates containing 0.1% TCA). The spores were incubated within the conditioned media for 2 h to allow triggering of germination. The samples were heated at 65°C for 20 min to kill any spores in which germination had been initiated. The number of non-germinated spores within the sample was then determined by plating the heated samples onto BHI agar containing 0.1% TCA. The relative percentage of germinating spores was calculated by subtracting the remaining spores from the initial spore count to provide an indication of the number of germinated vegetative cells in the population. The percentage of spores germinating was then calculated using the following equation

$$Percentage\;germination=\left(vegetative\;cells/initial\;inoculum\right)\;x100$$

### Amplification of BSH sequence

Consensus-degenerate hybrid oligonucleotide (Codehop) primers were designed as described previously.^54^ These primers amplify multiple BSH genes from microbiome samples using a single set of primers. Cycling conditions were 95°C for 2 min, followed by 30 cycles at 60°C with amplification at 75°C.^55^

### *Sequencing and genome assembly of* lactobacillus *strains*

A single colony of each *L. murinus* was inoculated into 20 ml of MRS broth and grown statically at 37°C for 24 h. DNA was extracted using a DNAEasy Blood and Tissue kit (Qiagen) as per the manufactures’ instructions. DNA was stored at −80°C until required. Genome sequencing was performed at Glasgow Polyomics facility (GPF). Low-quality sequences were trimmed and assembly was carried out using Spades assembly tool.^56^ Annotation was carried out using PROKKA.^57^

**Tax4Fun analysis**^30^ was used to find the metabolic function of the bacterial community by blasting OTUs against SILVA v115 database as all relevant pathways (KEGG database release 64.0) available. For this study, we recovered 6,297 KEGG orthologs (enzymes) using fctProfiling = TRUE in Tax4Fun() function. Although the Tax4Fun-based metabolic predictions are limited by the taxa available in the reference database, it provides a statistic called fraction-of-taxonomic-units-unexplained (FTU), which reflects the quantity of sequences that are assigned to a taxonomic unit but are not transferable to KEGG reference organisms. The summary statistics of FTUs in this study: *[Min: 0.004319; 1st Quartile:0.012234; Median: 0.020940; Mean: 0.028830; 3rd Quartile: 0.031007; Max: 0.137971]* revealed a very high ~91% mean coverage of taxa, increasing our confidence in the recovered metabolic pathways.

### Lactobacillus *BSH PCR*

Specific primers were designed to amplify the *Lactobacillus murinus* BSH using genomic sequence data (Supplementary Table 2). Cycling conditions were 95°C for 2 min, followed by 30 cycles at 50°C with amplification at 75°C

### Bile acid analysis and data processing

To determine the relative concentrations of bile acids including TCA, DCA, cholic acid, tauromuricholic acids and muricholic acids in the cecum of mice, Ultra Performance Liquid Chromatography-Quadrupole Time of Flight-Mass Spectrometry (UPLC-QToF-MS; UPLC Acquity, Waters Ltd.; Q-ToF Premier MS, Waters MS Technologies) was used. Cecal bile acid extracts (8 µl) were injected into a 2.1 × 100 mm (1.7 μm) HSS T3 Acquity column (Waters Corp., Milford, MA) and eluted using a linear gradient of 15% B to 95% B (A = water + 0.1% formic acid; B = acetonitrile + 0.1% formic acid) from 0 to 9 min and keeping for 1 min at 95% B followed by a 2 min column re-equilibration at 15% B. The quality control sample, formed by pooling a small portion from each sample, was injected every seven samples to check the instrumental stability. The MS data was recorded in negative electrospray mode with a scan range of 50–1000 m/z.^58^ The data was pre-processed using XC-MS^59^ package in R and the processed data was imported into SIMCA-P (Sartorius-stedium Biotech, Sweden) for principal component analysis and orthogonal projections to latent structures discriminant analysis.

### Statistical analysis

Statistical analysis was carried out in Graph Pad Prism and R version 8.1. The tests and parameters used are detailed in the figure legends throughout. Tests used included T-test with Welch correction, ANOVA with Tukey’s post-test and Kruskal–Wallis with Dunn’s multiple comparisons.

## Supplementary Material

Supplemental Material
