# Supplementary material for: Bile salt metabolism is not the only factor contributing to Clostridioides (Clostridium) difficile disease severity in the murine model of disease
Source: Gut Microbes. 2019 Dec 2;11(3):481–96. doi: 10.1080/19490976.2019.1678996 (PMC7524298; doi:10.1080/19490976.2019.1678996)
Supplement: Supplemental Material [file KGMI_A_1678996_SM0531.zip › Supplementary information/Supplementary Figure and Table legends.docx]

**Supplementary Figure and Table legends**

**Supplementary Figure S1; Bile salt metabolism.**

Pictorial representation of bile salt metabolism highlighting the role of the enzymes involved in modification of the bile acids. The structural changes that these enzymes facilitate, during the modification of the conjugated bile salt, taurocholate, to cholate and latterly to the secondary bile salt deoxycholate, are represented.

**Supplementary Figure S2; Enumeration of *C. difficile* in the caecum and colon of infected animals**

CFU per ml of vegetative cells (blue) and spores (red) present in the caecum and colon of A) mice treated with clindamycin only (n=10) B) Antibiotic cocktail plus clindamycin (n=10) and C) ABC alone (n=5). Error bars represent +/- the SEM. No bacteria were recovered from the untreated mice. Statistical analysis was performed using Kruskal Wallis test with Dunn’s comparison. This found that there were significantly more vegetative cells in association with the tissue in the caecum of the mice treated with the ABC and clindamycin (p= <0.5) although the impact of this is unclear.

**Supplementary Figure S3. Summary of the retention time and the mass to charge ratios of the detected bile acids.** The table shows a summary of the retention time and the mass to charge ratios of the detected bile acids in this study. Representative traces include A. a quality control trace generated from pooled samples from all study groups, B. a representative trace from animals treated with clindamycin alone, C a representative trace from mice treated with clindamycin + antibiotic cocktail and D. a representative trace from a mouse untreated with antibiotics.

**Supplementary Table 1**: **Differential analysis of OTUs between Clindamycin and ABC+Clindamycin at D0 (S4)**. For differential analysis, we have used DESeqDataSetFromMatrix( ) function from DESeq2 package. Briefly, in the method the negative binomial GLM is used to obtain maximum likelihood estimates for an OTU’s log-fold change between Clindamycin alone versus ABC + clindamycin treatment. Afterwards Bayesian shrinkage, using a zero-centred normal distribution as a prior, is used to shrink the log-fold change towards zero for those OTUs of lower mean count and/or with higher dispersion in their count distribution. These shrunken long fold changes are then used with the Wald test for significances. OTUs found to be discriminant after 2 log-fold change and Adjusted P value ≤ 0.05 are thus reported. OTUs upregulated in Clindamycin group are shaded as grey.
