# Supplementary material for: Bile salt metabolism is not the only factor contributing to Clostridioides (Clostridium) difficile disease severity in the murine model of disease
Source: Gut Microbes. 2019 Dec 2;11(3):481–96. doi: 10.1080/19490976.2019.1678996 (PMC7524298; doi:10.1080/19490976.2019.1678996)
Supplement: Supplemental Material [file KGMI_A_1678996_SM0531.zip › Supplementary information/Supplementary Table 1.docx]

**Supplementary Table 1**: **Differential analysis of OTUs between Clindamycin and ABC+Clindamycin at D0 (S4)**. For differential analysis, we have used DESeqDataSetFromMatrix( ) function from DESeq2 package. Briefly, in the method the negative binomial GLM is used to obtain maximum likelihood estimates for an OTU’s log-fold change between Clindamycin alone versus ABC + clindamycin treatment. Afterwards Bayesian shrinkage, using a zero-centered normal distribution as a prior, is used to shrink the log-fold change towards zero for those OTUs of lower mean count and/or with higher dispersion in their count distribution. These shrunken long fold changes are then used with the Wald test for significances. OTUs found to be discriminant after 2 log-fold change and Adjusted P value ≤

0.05 are thus reported. OTUs upregulated in Clindamycin group are shaded as grey.

| **Discriminant OTUs** | **Base mean abundance** | **Log-fold change** | **P value** | **Adjusted P value** | **Upregulated** |
| --- | --- | --- | --- | --- | --- |
| OTU_15 Bacteria;Firmicutes;Bacilli;Lactobacillales;Lactobacillaceae;Lactobacillus;Lactobacillus sp. M20 | 498.292278 | 7.16384504 | 1.39E-33 | 2.80E-31 | Clindamycin |
| OTU_11 Bacteria;Bacteroidetes;Bacteroidia;Bacteroidales;Porphyromonadaceae;Parabacteroides | 1060.40136 | -7.1530599 | 5.20E-20 | 5.22E-18 | ABC + Clindamycin |
| OTU_780 Bacteria;Bacteroidetes;Bacteroidia;Bacteroidales;Porphyromonadaceae;Parabacteroides;bacterium NLAE-zl-P241 | 2514.91276 | -11.264161 | 1.22E-19 | 8.14E-18 | ABC + Clindamycin |
| OTU_3 Bacteria;Bacteroidetes;Bacteroidia;Bacteroidales;Porphyromonadaceae;Parabacteroides | 12058.1062 | -9.2103843 | 4.70E-17 | 2.36E-15 | ABC + Clindamycin |
| OTU_2 Bacteria;Bacteroidetes;Bacteroidia;Bacteroidales;Bacteroidaceae;Bacteroides;Bacteroides thetaiotaomicron | 4460.70632 | -7.3714773 | 4.78E-16 | 1.92E-14 | ABC + Clindamycin |
| OTU_784 Bacteria;Bacteroidetes;Bacteroidia;Bacteroidales;Porphyromonadaceae;Parabacteroides | 395.276555 | -8.9027746 | 1.74E-14 | 5.82E-13 | ABC + Clindamycin |
| OTU_772 Bacteria;Bacteroidetes;Bacteroidia;Bacteroidales;Porphyromonadaceae;Parabacteroides;bacterium NLAE-zl-P743 | 307.046415 | -8.6652995 | 7.56E-14 | 2.17E-12 | ABC + Clindamycin |
| OTU_36 Bacteria;Bacteroidetes;Bacteroidia;Bacteroidales;S24-7 | 71.3237358 | 4.17403462 | 4.41E-13 | 1.11E-11 | Clindamycin |
| OTU_21 Bacteria;Proteobacteria;Gammaproteobacteria;Enterobacteriales;Enterobacteriaceae;Enterobacter;Enterobacter cloacae subsp. dissolvens | 13783.9259 | 10.3394326 | 4.98E-12 | 1.11E-10 | Clindamycin |
| OTU_34 Bacteria;Firmicutes;Clostridia;Clostridiales;vadinBB60 | 546.064125 | -7.4092695 | 7.81E-12 | 1.57E-10 | ABC + Clindamycin |
| OTU_233 Bacteria;Firmicutes;Bacilli;Lactobacillales;Streptococcaceae;Lactococcus;Lactococcus garvieae | 130.108094 | 7.85374743 | 1.01E-11 | 1.84E-10 | Clindamycin |
| OTU_45 Bacteria;Firmicutes;Bacilli;Lactobacillales;Lactobacillaceae;Lactobacillus | 126.805241 | 5.67238533 | 2.69E-10 | 4.38E-09 | Clindamycin |
| OTU_91 Bacteria;Deferribacteres;Deferribacteres;Deferribacterales;Deferribacteraceae;Mucispirillum | 77.721083 | 4.77541855 | 2.83E-10 | 4.38E-09 | Clindamycin |
| OTU_22 Bacteria;Firmicutes;Erysipelotrichia;Erysipelotrichales;Erysipelotrichaceae;Allobaculum | 1270.59888 | -7.6979999 | 6.06E-10 | 8.69E-09 | ABC + Clindamycin |
| OTU_812 Bacteria;Bacteroidetes;Bacteroidia;Bacteroidales;Porphyromonadaceae;Parabacteroides | 86.8645752 | -7.3614466 | 1.02E-09 | 1.37E-08 | ABC + Clindamycin |
| OTU_468 Bacteria;Bacteroidetes;Bacteroidia;Bacteroidales;Bacteroidaceae;Bacteroides | 66.0156105 | -4.8448564 | 3.44E-09 | 4.11E-08 | ABC + Clindamycin |
| OTU_24 Bacteria;Bacteroidetes;Bacteroidia;Bacteroidales;Bacteroidaceae;Bacteroides;bacterium NLAE-zl-H49 | 320.763109 | -6.8401164 | 3.48E-09 | 4.11E-08 | ABC + Clindamycin |
| OTU_850 Bacteria;Bacteroidetes;Bacteroidia;Bacteroidales;Porphyromonadaceae;Parabacteroides | 51.4562293 | -6.6013093 | 8.52E-09 | 9.51E-08 | ABC + Clindamycin |
| OTU_7 Bacteria;Firmicutes;Bacilli;Lactobacillales;Enterococcaceae;Enterococcus;Enterococcus casseliflavus | 3764.25423 | -7.4336229 | 1.02E-08 | 1.07E-07 | ABC + Clindamycin |
| OTU_5 Bacteria;Firmicutes;Erysipelotrichia;Erysipelotrichales;Erysipelotrichaceae;Allobaculum | 160.358588 | -4.6448047 | 1.45E-08 | 1.46E-07 | ABC + Clindamycin |
| OTU_10 Bacteria;Bacteroidetes;Bacteroidia;Bacteroidales;S24-7 | 504.470958 | -5.1054646 | 3.99E-08 | 3.82E-07 | ABC + Clindamycin |
| OTU_181 Bacteria;Actinobacteria;Coriobacteriia;Coriobacteriales;Coriobacteriaceae;Enterorhabdus | 17.3964113 | 3.2462064 | 1.77E-06 | 1.62E-05 | Clindamycin |
| OTU_40 Bacteria;Bacteroidetes;Bacteroidia;Bacteroidales;S24-7 | 37.4057691 | -4.3025763 | 3.04E-06 | 2.65E-05 | ABC + Clindamycin |
| OTU_53 Bacteria;Bacteroidetes;Bacteroidia;Bacteroidales;S24-7 | 31.5167453 | 3.20402736 | 3.80E-06 | 2.94E-05 | Clindamycin |
| OTU_17 Bacteria;Proteobacteria;Betaproteobacteria;Burkholderiales;Alcaligenaceae;Parasutterella | 806.8003 | -4.4125039 | 3.73E-06 | 2.94E-05 | ABC + Clindamycin |
| OTU_122 Bacteria;Firmicutes;Clostridia;Clostridiales;vadinBB60 | 33.6316215 | -5.024464 | 3.64E-06 | 2.94E-05 | ABC + Clindamycin |
| OTU_47 Bacteria;Bacteroidetes;Bacteroidia;Bacteroidales;Porphyromonadaceae;Odoribacter | 25.2277815 | 2.68833089 | 6.58E-06 | 4.90E-05 | Clindamycin |
| OTU_88 Bacteria;Firmicutes;Clostridia;Clostridiales;Lachnospiraceae;Incertae Sedis | 12.4733345 | 3.19938069 | 1.03E-05 | 7.24E-05 | Clindamycin |
| OTU_733 Bacteria;Bacteroidetes;Bacteroidia;Bacteroidales;Porphyromonadaceae;Parabacteroides | 16.9878918 | -4.9721955 | 1.04E-05 | 7.24E-05 | ABC + Clindamycin |
| OTU_755 Bacteria;Bacteroidetes;Bacteroidia;Bacteroidales;Porphyromonadaceae;Parabacteroides | 17.2762032 | -4.9971521 | 1.11E-05 | 7.42E-05 | ABC + Clindamycin |
| OTU_750 Bacteria;Bacteroidetes;Bacteroidia;Bacteroidales;Porphyromonadaceae;Parabacteroides | 16.9597427 | -4.969284 | 1.52E-05 | 9.86E-05 | ABC + Clindamycin |
| OTU_734 Bacteria;Bacteroidetes;Bacteroidia;Bacteroidales;Porphyromonadaceae;Parabacteroides | 17.6270182 | -5.0263337 | 1.59E-05 | 0.00010003 | ABC + Clindamycin |
| OTU_64 Bacteria;Proteobacteria;Betaproteobacteria;Burkholderiales;Alcaligenaceae;Parasutterella | 124.481368 | -5.2880639 | 1.85E-05 | 0.0001128 | ABC + Clindamycin |
| OTU_756 Bacteria;Bacteroidetes;Bacteroidia;Bacteroidales;Porphyromonadaceae;Parabacteroides | 15.2724756 | -4.8131205 | 2.61E-05 | 0.00015438 | ABC + Clindamycin |
