# Supplementary material for: Bile salt metabolism is not the only factor contributing to Clostridioides (Clostridium) difficile disease severity in the murine model of disease
Source: Gut Microbes. 2019 Dec 2;11(3):481–96. doi: 10.1080/19490976.2019.1678996 (PMC7524298; doi:10.1080/19490976.2019.1678996)
Supplement: Supplemental Material [file KGMI_A_1678996_SM0531.zip › Supplementary information/Supplementary Table 2.docx]

| **Name** | **Description** | **Sequence** |
| --- | --- | --- |
| BSH G1 (F) | Degenerate primers for amplification of BSH.  Primers provided by Prof Julian Marchesi | CGTCCAGGCCCGGACNATHGARTGGG |
| BSH G1 (R) |  | GGTGGCGGAGGTCCAYTGNGT |
| 16s rRNA V4 (F) | Primers for amplification of the V4 region of the 16s rRNA gene. The forward primer is universal. The reverse primer contains a unique 12bp Golay barcode (bold) to allow multiplexing of samples for sequencing  Primers kindly provided by Prof Chris Quince | AATGATACGGCGACCACCGAGATCTACACTATGGTAATTGT  GTGNCAGCMGCCGCGGTAA |
| 16s rRNA V4 (R) |  | CAAGCAGAAGACGGCATACGAGAT-**GTCGAATTTGCG-**AGTCAGTCAGCCGGACTACHVGGGTWTCTAAT |
| S12tBSH | Production of both the truncated BSH from  *Lactobacillus murinus* S12 and the full length BSH from S15 | GGGAGAAATTTTGACTATGAG |
| S15BSH |  | ATGTGTACTGCAGTATCATTTAAAAC |
| BSH R |  | TTAGTTAGCGTAATTGAAATG |

**Supplementary Table 2: Primers used during this study.**
