# Supplementary material for: Bile salt metabolism is not the only factor contributing to Clostridioides (Clostridium) difficile disease severity in the murine model of disease
Source: Gut Microbes. 2019 Dec 2;11(3):481–96. doi: 10.1080/19490976.2019.1678996 (PMC7524298; doi:10.1080/19490976.2019.1678996)
Supplement: Supplemental Material [file KGMI_A_1678996_SM0531.zip › Supplementary information/Supplementay Figure 3 .pptx]

## Slide 1
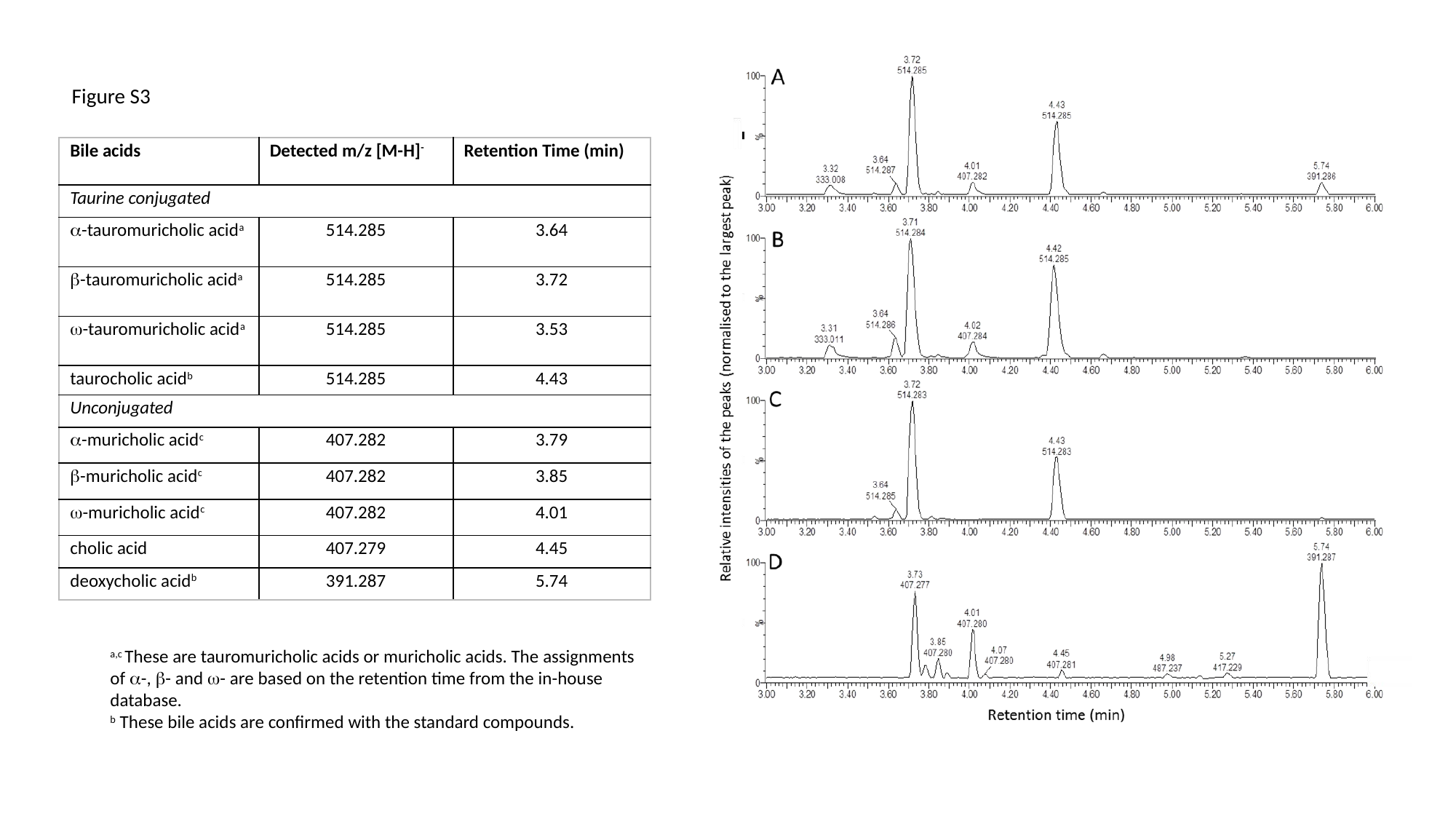

Figure S3
| Bile acids | Detected m/z [M-H]- | Retention Time (min) |
| --- | --- | --- |
| Taurine conjugated | | |
| a-tauromuricholic acida | 514.285 | 3.64 |
| b-tauromuricholic acida | 514.285 | 3.72 |
| w-tauromuricholic acida | 514.285 | 3.53 |
| taurocholic acidb | 514.285 | 4.43 |
| Unconjugated | | |
| a-muricholic acidc | 407.282 | 3.79 |
| b-muricholic acidc | 407.282 | 3.85 |
| w-muricholic acidc | 407.282 | 4.01 |
| cholic acid | 407.279 | 4.45 |
| deoxycholic acidb | 391.287 | 5.74 |
a,c These are tauromuricholic acids or muricholic acids. The assignments of a-, b- and w- are based on the retention time from the in-house database.
b These bile acids are confirmed with the standard compounds.
