# Supplementary figures and images for: Bile salt metabolism is not the only factor contributing to Clostridioides (Clostridium) difficile disease severity in the murine model of disease
Source: Gut Microbes. 2019 Dec 2;11(3):481–96. doi: 10.1080/19490976.2019.1678996 (PMC7524298; doi:10.1080/19490976.2019.1678996)

## Slide 1
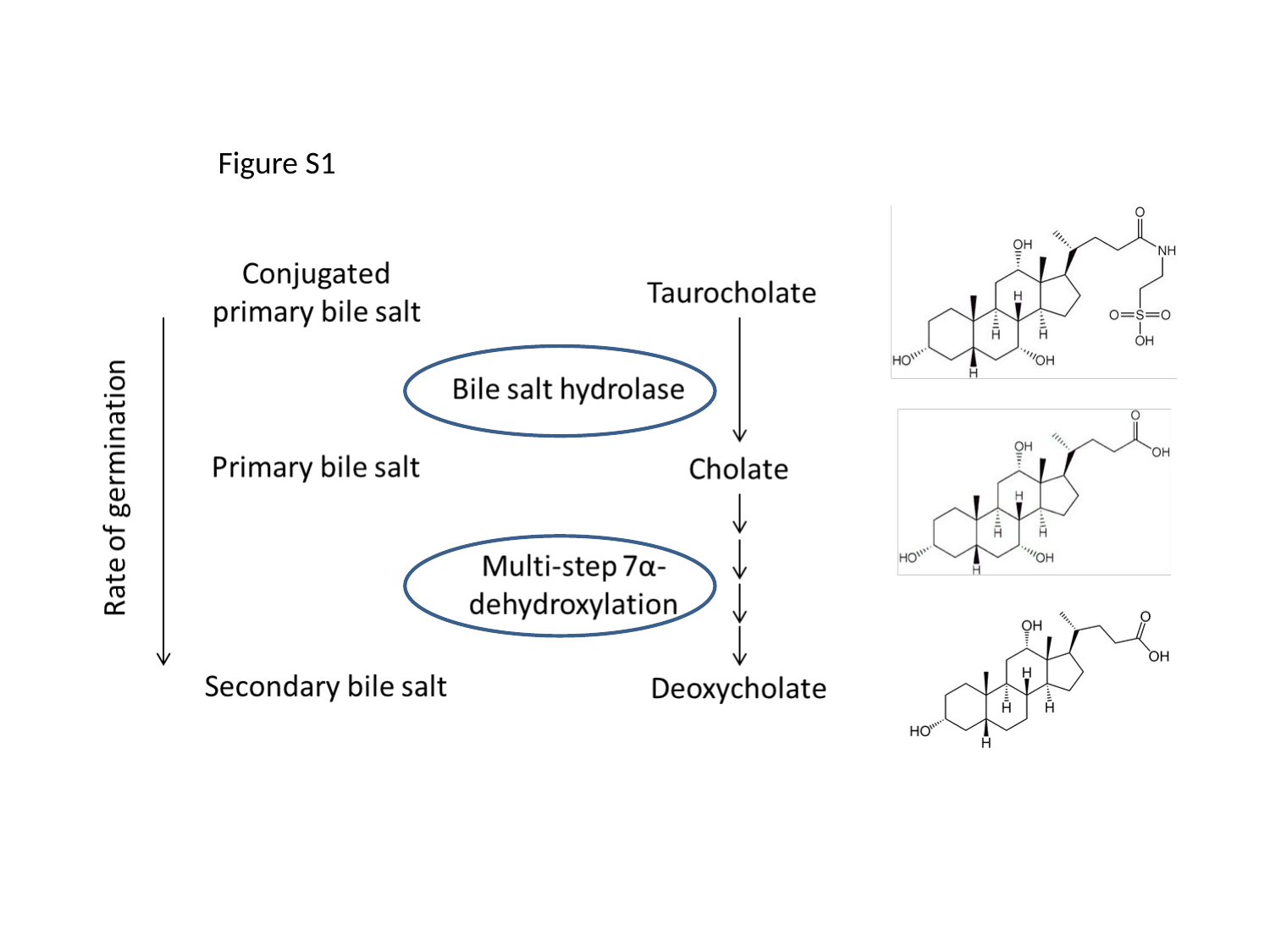

Figure S1

Supplement: Supplemental Material [file KGMI_A_1678996_SM0531.zip › Supplementary information/supplementary figure 1.pptx]

## Slide 1
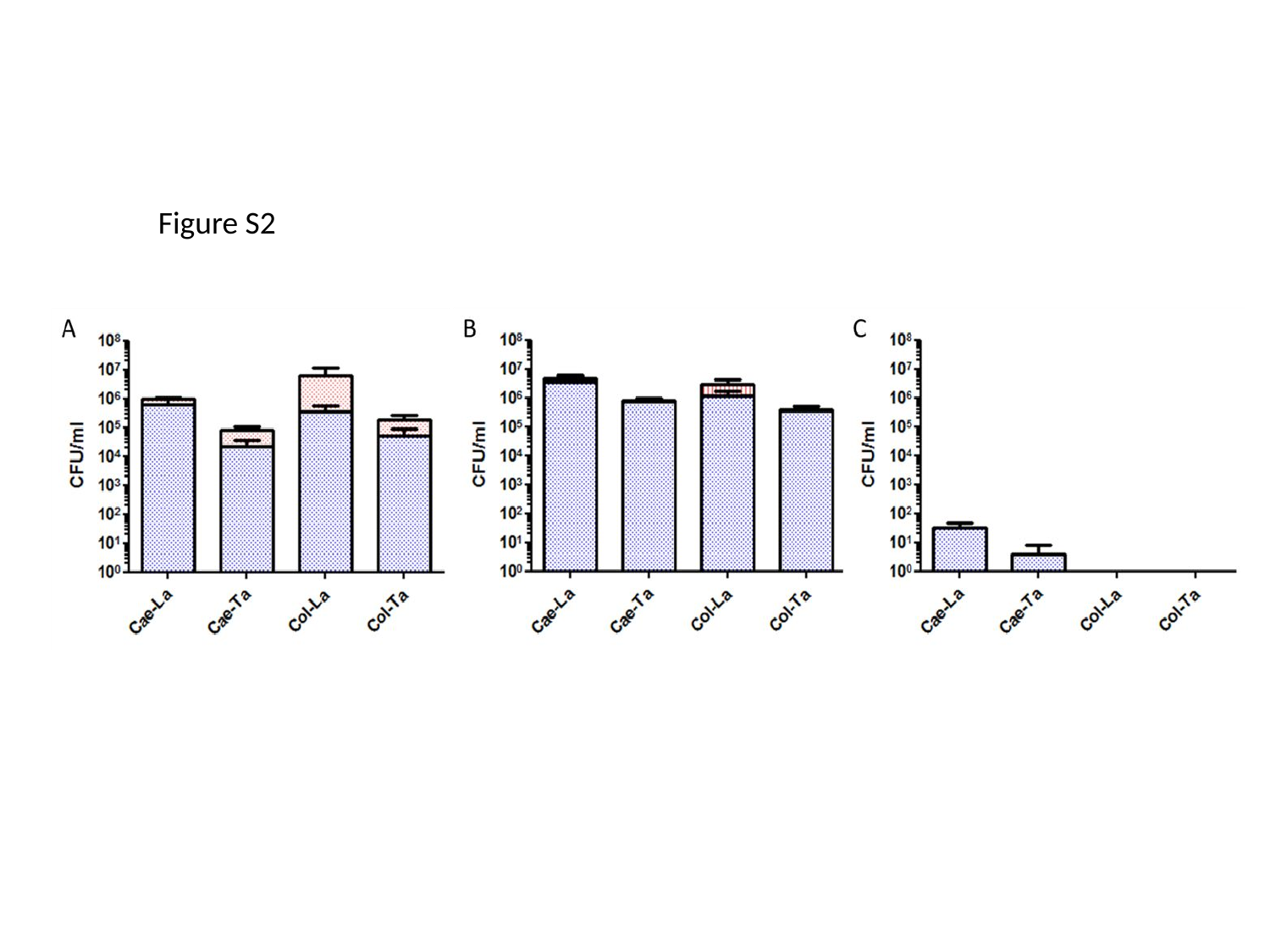

Figure S2

Supplement: Supplemental Material [file KGMI_A_1678996_SM0531.zip › Supplementary information/supplementary Figure 2.pptx]
